# Fostering local production of essential medicines in Nigeria

**DOI:** 10.2471/BLT.19.249508

**Published:** 2020-06-02

**Authors:** Omotayo Fatokun

**Affiliations:** a Faculty of Pharmaceutical Sciences, UCSI University, No. 1, Jalan Menara Gading, UCSI Heights, Cheras 56000 Kuala Lumpur, Malaysia.

Consistent availability and access to medicines in low- and middle-income countries, particularly in sub-Saharan Africa, is a challenge. As a result, the governments in these countries have shown increasing interest in local pharmaceutical production as a means of promoting technology transfer, building capacity and improving access to essential medicines.^1^ In 2019, the National Agency for Food and Drug Administration and Control in Nigeria introduced the Five Plus Five-Year Validity (Migration to Local Production) policy,^2^ in support of producing essential medicines locally. In line with this policy, as of 1 May 2019, a newly registered imported product is given a maximum period of 10 years (five years of initial registration plus another five years of renewal registration) to migrate to local production. 

This transition can be made through a partnership with a Nigerian company and/or setting up a local manufacturing plant in Nigeria for finished pharmaceutical products, active pharmaceutical ingredients, non-active pharmaceutical ingredients or packaging materials, among others. Failing to do so would cancel the product’s registration,^2^ preventing its importation and distribution in Nigeria. Similarly, existing registered imported products that have had their registration renewed more than twice were required to provide evidence of a migration plan to local manufacturing by 31 December 2019, which should be implemented within four years of being submitted.^2^ Migration to local manufacturing is limited to those products that local manufacturers have the capacity to produce or to those that the new partnership would enhance the capacity to manufacture.^2^

The main aims of the policy are to reduce the number of pharmaceutical products imported into Nigeria and encourage local production of essential medicines.^3^ Approximately 70% of drugs consumed in Nigeria (that is, pharmaceutical dosage forms-tablets, capsules, syrups and ointments, among others), are imported.^4^ Therefore, the policy will help increasing the capacity utilization of local manufacturing facilities, which in 2011 were operating at around 40% of their capacity.^4^ A 2005 government ban (still in effect) on imports of 18 essential medicines for which there was adequate domestic production capacity and technical skills to produce, resulted in an increase of average annual local production levels in solid dosage forms from about 15% to 40%.^4^ Compared to the 2005 ban, the current policy has a greater potential for success, because it is broader in scope, includes a much wider range of pharmaceutical formulations, is more strategic and involves direct engagement between the government, local producers and potential foreign investors. However, one of the main reasons for the low capacity utilization in the pharmaceutical manufacturing sector in Nigeria is the lack of competitiveness with imported products.^4^ Therefore, the government needs to provide a customized package of fiscal and non-fiscal incentives to facilitate local pharmaceutical production at a competitive price.^1^^,^^5^

The fiscal policy measure implemented since 2016 reduced the import adjustment tax under the Economic Community of West African States Common External Tariff on pharmaceutical raw materials from 5–20% to 0% and imposed a 20% import adjustment tax on four groups of imported drugs that can be produced by local manufacturers, including antimalarials, antibiotics, alkaloid derivatives and vitamins.^6^^,^^7^ The measure is seen as complementary to the 2019 policy. A 2017 Federal Executive Order stipulating that at least 40% of the government’s procurement expenditure be locally manufactured goods or local services is also encouraging.^8^ However, other measures are needed, such as grants, subsidies, soft loans (that is, with no interest or below-market rates of interest, and with longer repayment periods than conventional loans), development of robust policy structures in government ministries and agencies involved in health, creation of an investment-friendly environment and infrastructure development.^1^^,^^6^ Nigerian pharmaceutical manufacturers recently called on the government to establish a pharmaceutical expansion and export fund that would provide soft loans to upgrade and improve factories, as well as increase capacity among local drug manufacturing companies.^8^ Moreover, since Nigeria imports practically all the ingredients needed for its local pharmaceutical production, government support for the growth of the active pharmaceutical ingredients industry would be crucial to the long-term sustainability of local production.^8^

Furthermore, local pharmaceutical production can help to shorten the supply chain and can contribute to alleviating the problems of shortages and stock-outs of essential medicines,^9^ ensuring reliable and timely access to needed medicines. With increased local production, Nigeria’s high dependence on imported products can be reduced, decreasing the influx of substandard and falsified medicines into the country. An estimated 17% of total pharmaceutical market (in volume) included substandard and falsified medicines.^3^ Many of these medicines are imported as unregistered or are registered but compromised on the content after approval, becoming substandard.^3^

While local pharmaceutical production in some low-income countries is not viable because of limited local technical expertise or low economies of scale,^10^ this issue may not be the case in Nigeria. According to a United Nations Industrial Development Organization report, “in Nigeria a population of over 140 million [now over 190 million] represents a huge potential market and local expertise and experience in the manufacture of essential medicines exist… the commercial prospects for local drug manufacture are positive.”^4^

The potential for positive effects of the new policy in Nigeria could be drawn from the results of similar policy initiatives in Bangladesh, a country with a population similar to that of Nigeria. In 1982, the Government of Bangladesh introduced the National Drug Policy and the Drug Control Ordinance.^11^ The key objectives of the policy were to promote local pharmaceutical production, reduce the cost of pharmaceuticals and remove harmful or inefficient products from the market. The Drug Control Ordinance banned the sale of certain medicines, limited pharmaceuticals from being imported if foreign firms did not have a manufacturing plant in Bangladesh or if the drug or its equivalent was already produced in-country, and allowed the government to set drug prices.^11^ The introduction of market incentives (such as financial support for the promotion of industrial growth through preferential loan approvals and better interest rates and value-added tax exemption for local produced products), combined with the policy reforms introduced by both the policy and ordinance, became a catalyst for increased investment and development in Bangladesh’s local pharmaceutical industry.^11^ These initiatives have resulted in a significant drop in drug importation, and the domestic industry now meets 98% of local demand for essential medicines and exports to 151 countries.^11^

Coupled with appropriate fiscal and non-fiscal incentives, the new policy in Nigeria can therefore contribute to increasing local pharmaceutical production and to developing the domestic pharmaceutical industry. However, studies from Ethiopia and United Republic of Tanzania (conducted as background situation analyses before implementing local production plans) suggest that supporting local drug manufacturers through fiscal and/or non-fiscal incentives must have a specified timeframe, and be developed and executed in a transparent manner, so patients do not end up paying higher drug prices.^12^ The studies found that lack of price competitiveness of locally produced medicines would be a barrier to the growth of domestic industry and made suggestions on how to enhance a more competitive local production.^12^

Furthermore, systems for monitoring the availability and prices of locally produced and imported medicines on a regular basis need to be established to assess the impact of local production on access to medicines.^12^ The policy in Nigeria recognizes that not all products can be manufactured locally and therefore, some products might have to be imported.^2^

Beyond promoting local pharmaceutical production, Nigeria should keep enhancing its regulatory oversight of pharmaceutical products and ensure strict compliance with the required quality control systems in accordance with acceptable international quality standards for pharmaceutical production. Indeed, no local production of medical products is desirable without quality assurance.^1^ Therefore, strong regulatory capacity is essential to increase confidence in the quality, efficacy and safety of locally produced medicines in Nigeria. Particularly, enhanced regulatory capacity in the inspection of drug manufacturing facilities to meet international Good Manufacturing Practice standards, enforcement of regulation and post marketing surveillance is important. In this respect, continued capacity building and technical support from World Health Organization and other international partners is needed. The new regulatory policy to increase local production of essential medicines in Nigeria should contribute to ensuring predictable and sustainable access to affordable and quality-assured essential medicines, and strengthening the domestic pharmaceutical industry.
